# Creating Anti-icing Surfaces via the Direct Immobilization of Antifreeze Proteins on Aluminum

**DOI:** 10.1038/srep12019

**Published:** 2015-07-08

**Authors:** Yunho Gwak, Ji-in Park, Minjae Kim, Hong Suk Kim, Myong Jong Kwon, Seung Jin Oh, Young-Pil Kim, EonSeon Jin

**Affiliations:** 1 Department of Life Science, Research Institute for Natural Science, Hanyang University, Seoul, 133-791, South Korea; 2 DMC R&D Center, Samsung Electronics co. Ltd., (Meatan dong) 129, Samsung-ro Yeongtong-gu, Suwon-si, Gyeonggi-do 443-742, South Korea; 3 Institute of Nano Science and Technology, Research Institute for Convergence of Basic Sciences, Hanyang University, Seoul, 133-791, South Korea

## Abstract

Cryoprotectants such as antifreeze proteins (AFPs) and sugar molecules may provide a solution for icing problems. These anti-icing substances protect cells and tissues from freezing by inhibiting ice formation. In this study, we developed a method for coating an industrial metal material (aluminum, Al) with AFP from the Antarctic marine diatom, *Chaetoceros neogracile* (*Cn*-AFP), to prevent or delay ice formation. To coat Al with *Cn*-AFP, we used an Al-binding peptide (ABP) as a conjugator and fused it with *Cn*-AFP. The ABP bound well to the Al and did not considerably change the functional properties of AFP. *Cn*-AFP-coated Al (*Cn*-AFP-Al) showed a sufficiently low supercooling point. Additional trehalose coating of *Cn*-AFP-Al considerably delayed AFP denaturation on the Al without affecting its antifreeze activity. This metal surface–coating method using trehalose-fortified AFP can be applied to other metals important in the aircraft and cold storage fields where anti-icing materials are critical.

Ice formation is a major problem in industries and applications such as air conditioners, transportation (including aircraft), and power generation^1^,^2^,^3^,^4^, because it reduces the cold resistance of equipment, leading to high energy loss^4^. To resolve this problem, surface-coating techniques based on thermal, chemical, and mechanical methods have been implemented to attain anti-icing properties; however, most of these rely on complicated processes that require expensive equipment and labor-intensive procedures^4^. To this end, new cryoprotectants that can be used as effective anti-icing materials have drawn the attention of the food, plant, military, and electronics industries. Whereas several cryoprotectants, such as glycols, polyols including glycerol, and sugars have been used, antifreeze proteins (AFPs) produced by psychrophilic organisms under freezing conditions^5^ have been recognized as one of the most exciting materials for use in various industrial and biological fields including food, medical, and cryopreservation^6^,^7^,^8^,^9^,^10^, due to their distinct ability to inhibit ice or frost formation in a concentration-independent manner^11^,^12^.

It is important to note that AFPs have been used to coat glass surfaces via polymer-linked conjugation to prevent ice formation^13^. Although the polymer-coating method can be used to improve the stability of AFPs, this strategy is generally limited to surface-bound proteins, because chemical modification of the surfaces is labor-intensive.

Aluminum (Al) is the third most abundant element in earth’s crust and has many properties that make it useful in a wide range of industrial fields including the transportation, construction, electronics, freezer and cryostat industries^14^. Al surfaces with anti-icing properties are of particular interest, because the frost that forms on Al-coated compressors in the outdoor units of air conditioners at cool temperatures causes critical damage to air circulation, which is inevitably accompanied by high energy consumption to remove the ice or frost^1^,^4^. To date, no viable anti-icing Al surfaces have been created. Given the significant anti-icing potential of AFPs, it is reasonable to expect that a robust method for AFP immobilization on an Al surface will provide new opportunities for producing an anti-icing surface.

Here we demonstrate the simple and robust fabrication of anti-icing Al surfaces using AFPs. We chose the AFP from the Antarctic marine diatom *Chaetoceros neogracile* (*Cn-*AFP). Not only can this protein be produced in large amounts via expression in *Escherichia coli*, it also exhibits great potential to lower the freezing point at a relatively low concentration in comparison with other AFPs^15^,^16^,^17^. In the present study, we introduce metal binding peptides fused to an AFP to effectively adsorb the *Cn-*AFP on Al surfaces, which allows for rapid immobilization of AFPs on the metal substrate. An Al-binding peptide (ABP) was fused to the N-terminus of *Cn*-AFP. This step was followed by a simple sugar-coating process to reinforce the protein stability. Since trehalose, a nonreducing sugar composed of two glucose residues, effectively retains water molecules^18^,^19^,^20^,^21^, it helps proteins maintain their conformation under dehydration conditions. Consequently, this simple immobilization of AFP followed by coating with trehalose allows for the generation of a long-lasting, anti-icing coating on a surface. To investigate the advantages of this strategy, we examined the anti-icing effects by measuring thermal hysteresis and the supercooling point of AFP-coated Al surfaces, and compared their properties with those of hydrophilic metal oxide coated Al surfaces, which are generally used in the outdoor air conditioners.

## Results

### AFP as a novel anti-icing material

**Figure 1:**
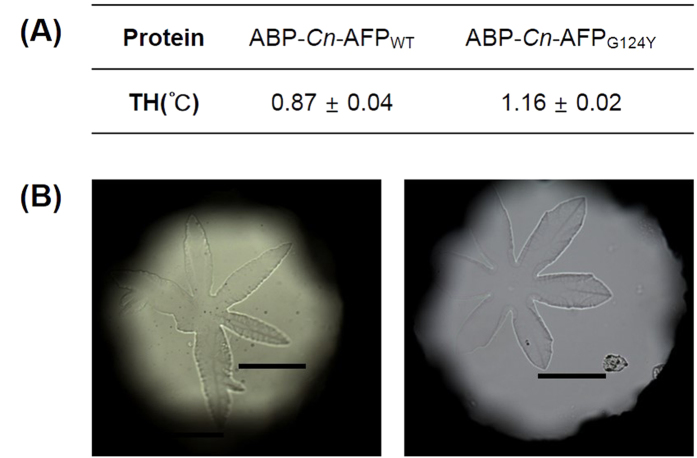
Antifreeze activities of ABP-*Cn*-AFP proteins. (**A**) Thermal hysteresis (TH) values of ABP-*Cn*-AFP_WT_ and ABP_G124Y_. (**B**) Ice crystal morphology in the presence of ABP-*Cn*-AFP_WT_ and ABP-*Cn*-AFP_G124Y_. The ice crystals showed burst patterns in the presence of both proteins. Scale bars: 100 μm.

**Figure 2:**
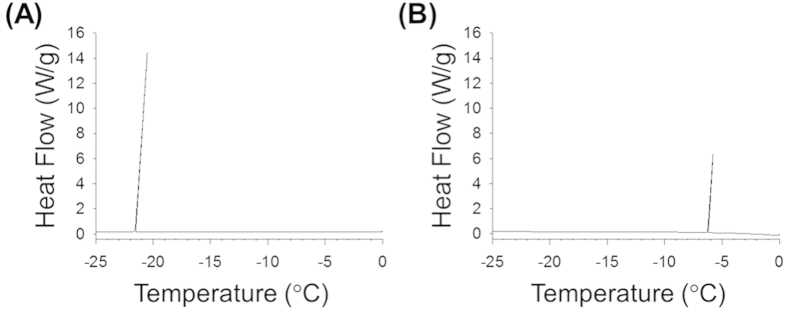
Determination of the supercooling point of ABP-*Cn*-AFP_G124Y_. Water was used as a negative control. Measurements were performed using a differential scanning calorimeter. The supercooling points of ABP-*Cn*-AFP_G124Y_ (**A**) and water (**B**) were −20.5 °C and −5.79 °C, respectively. Surface density of ABP-*Cn*-AFP_G124Y_ on the sample stage was 8 pmol/mm^2^.

To produce aluminum-binding *Cn*-AFP, we fused its N-terminus with an ABP sequence (VPSSGPQDTRTT), which was previously identified via the phage display method^22^. Since *Cn*-AFP allows the diatom to survive under freezing conditions by lowering the freezing point via ice binding^12^,^15^, we used wild-type *Cn*-AFP (*Cn*-AFP_WT_) and its mutant *Cn*-AFP_G124Y_, and their ABP-fused forms (ABP-*Cn*-AFP_WT_ and ABP-*Cn*-AFP_G124Y_). *Cn*-AFP_G124Y_ has been reported to exhibit a thermal hysteresis (TH) value 1.5 times higher than that of *Cn*-AFP_WT_^16^. This means a wider temperature gap between the melting and freezing points in the presence of *Cn*-AFP_G124Y_ than in the presence of *Cn*-AFP_WT_, due to the enhanced ability of *Cn*-AFP_G124Y_ to lower the freezing point of a liquid in a non-colligative way. To check whether ABP-fused *Cn*-AFPs retain their antifreeze activities, we assessed their effects on TH and ice crystal morphology (Fig. 1). We observed a slight reduction in the TH values of ABP-*Cn*-AFP_WT_ (0.9 °C) and ABP-*Cn*-AFP_G124Y_ (1.2 °C) in comparison with the reported values (1.2 °C and 2.0 °C, respectively)^16^. The ice crystals exhibit burst patterns in the presence of both proteins, appearing when a hyperantifreeze protein is present in the solution^23^. To further verify the antifreeze activity of ABP-*Cn*-AFP, the supercooling point of ABP-*Cn*-AFP_G124Y_ was measured via differential scanning calorimetry (Fig. 2). The supercooling point of ABP-*Cn*-AFP_G124Y_ was −20.5 °C, whereas that of water (negative control) was −5.79 °C. Taking into consideration that the supercooling point of AFP from the beetle *Dendroides canadensis* ranges from −16 to −26 °C^24^, this result indicates that ABP-*Cn*-AFP_G124Y_, despite a reduction in its TH value, has antifreeze activity.

### Construction and characterization of surface-immobilized AFPs on Al

**Figure 3:**
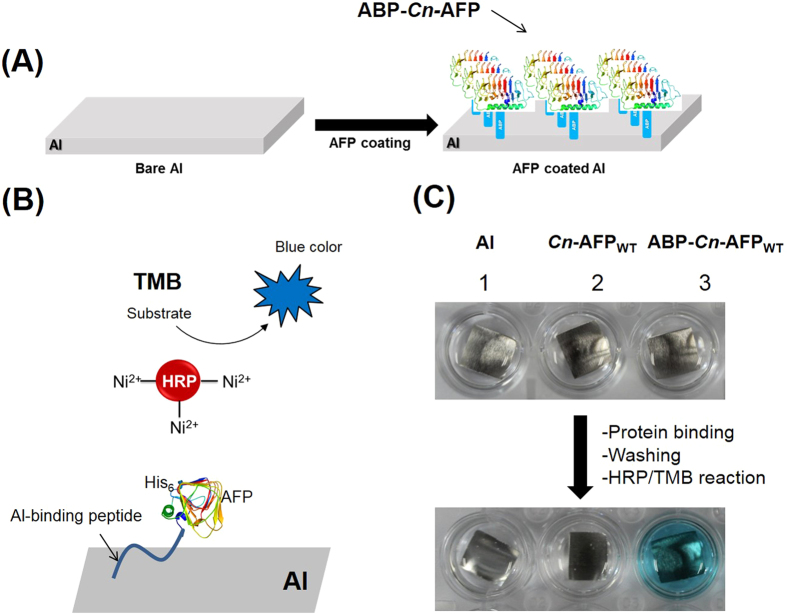
Coating of the Al substrate with *Cn*-AFP. (**A**) Scheme of Al coating with *Cn*-AFP. The blue corresponds to the Al-binding peptide. (**B**) Scheme of the tetramethylbenzidine (TMB) assay. TMB is a soluble colorimetric substrate for horseradish peroxidase (HRP). In the presence of HRP, TMB and peroxide present in the substrate solution react to produce a blue product. The color intensity is proportional to the HRP activity. (**C**) Confirmation of ABP-*Cn*-AFP binding to an Al substrate using the TMB assay. Al alone or after “coating” with *Cn*-AFP (without ABP) did not result in blue coloration. In the presence of ABP-*Cn*-AFP-coated Al, the solution color changed to blue, which indicated ABP-*Cn*-AFP binding to the Al.

**Figure 4:**
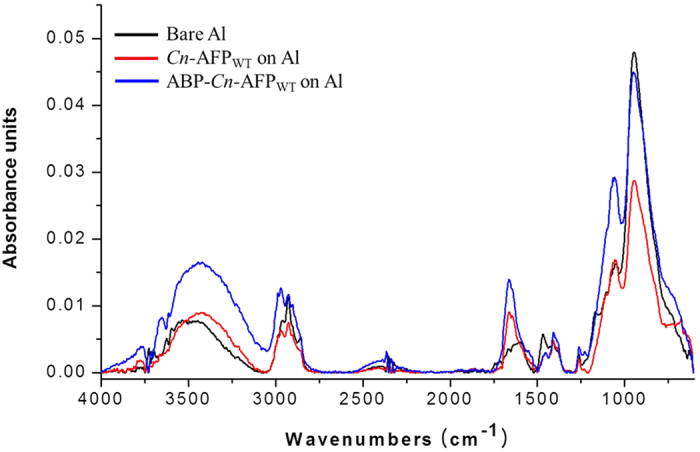
FT-IR spectra of three different Al surfaces. Bare Al (black line) and *Cn*-AFP-coated Al (red line) substrates were compared with ABP-*Cn*-AFP-coated Al (blue line). Black and red lines were used as the control surfaces, there was the difference in FT-IR spectra especially at the amide region (~1654 cm^−1^), where the red line was rather similar to the blue line (ABP-*Cn*-AFP_WT_ on Al).

To determine whether ABP-*Cn*-AFP can be effectively immobilized on the Al surface, a simple coating method was developed based on dipping an Al plate into a reaction solution containing *Cn*-AFP_WT_ or ABP-*Cn*-AFP_WT_ (Fig. 3A). The immobilization of AFP on Al was monitored using a colorimetric assay, where *Cn*-AFP_WT_ or ABP-*Cn*-AFP_WT_ expressed with His_6_-tag at its N-terminus was detected by Ni (II)/horseradish peroxidase (HRP), which produced a strong blue coloration because of HRP-catalyzed oxidation of its substrate (tetramethylbenzidine, TMB) (Fig. 3B). As shown in Fig. 3C, a strong blue color was significantly observed on the Al surface coated with ABP-*Cn*-AFP_WT_, while bare Al and ABP-free *Cn*-AFP_WT_ produced no coloration. To further verify the ABP-mediated binding of *Cn*-AFP to Al, three surfaces (bare Al, *Cn*-AFP_WT_ immobilized on Al, and ABP-*Cn*-AFP_WT_ immobilized on Al) were characterized via Fourier transform-infrared (FT-IR) spectroscopy (Fig. 4). While the Al−OH regions were commonly observed for all three tested surfaces, as indicated by three bands in the FT-IR spectra at ~955, ~1033, and 3535–3580 cm^−1^, the intensities of protein-specific peaks corresponding to N-H stretching (3700–3500 cm^−1^), amide C = O stretching (1690–1630 cm^−1^), and amide C-N stretching (1000–1250 cm^−1^) were higher for ABP-*Cn*-AFP_WT_ immobilized on the Al surface (blue line in Fig. 3) than those for the other two surfaces (black and red lines in Fig. 4). However, the red line (AFP without ABP tag) also showed an amide bond peak, which is presumed to be denatured after surface adsorption, taking consideration into Fig. 3. Taken together, these results indicate that ABP-fused *Cn*-AFP effectively binds to the Al surface via ABP without denaturation.

### Anti-icing surfaces via trehalose-coated AFPs on Al

**Figure 5:**
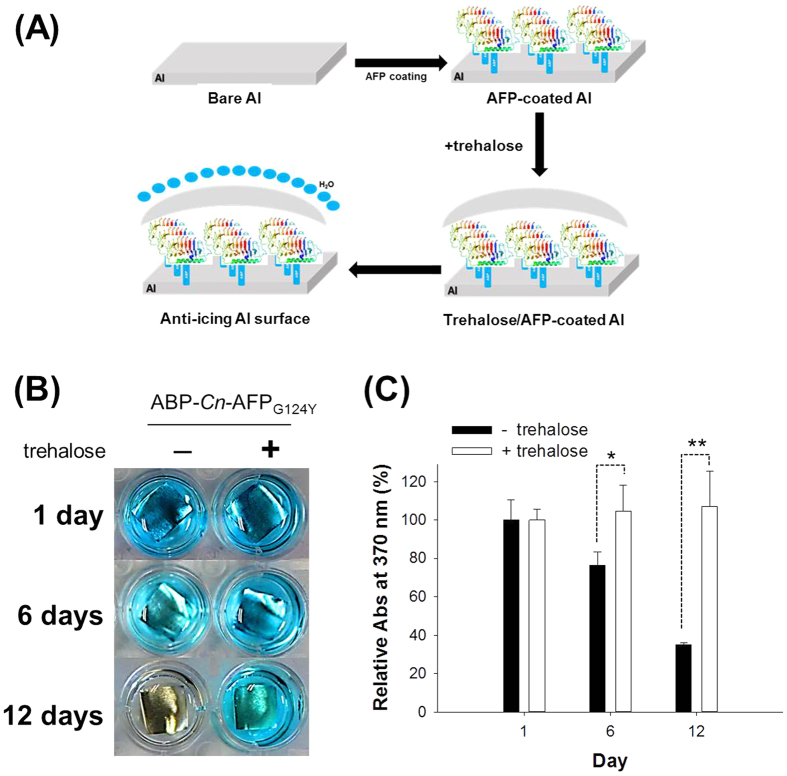
Secondary trehalose coating of ABP-*Cn*-AFP_G124Y_ on an Al plate. (**A**) Schematic diagram of trehalose coating procedure. (**B**) The TMB assay, six days after coating, the proteins were denatured in the absence of trehalose coating. Secondary trehalose coating preserved the protein structure. (**C**) Spectrophotometry of the TMB solutions. Without trehalose coating, proteins on the Al plate were denatured after 6 days. In the presence of trehalose coating, the protein was still not denatured twelve days after coating. Asterisks (* and **) denote statistical significance of the differences in colorimetric intensity of surface-immobilized ABP-*Cn*-AFP_G124Y_ with and without trehalose over the time course (*p* < 0.01, paired *t*-test, *n* = 4). Error bars indicate standard deviation.

Despite successful immobilization, denaturing of the AFP on the Al surface may reduce its antifreeze ability. To gain insight into AFP denaturation, we examined the stability of surface-immobilized *Cn*-AFP over time using the HRP/TMB colorimetric assay. We employed the trehalose-coating method to prevent protein denaturation, because trehalose is reported to make proteins resistant to dehydration when dried. Protein conformation can be preserved during drying through the hydrogen-bonded interaction of trehalose and water molecules, which efficiently competes with the interaction between protein and water molecules inside a trehalose-entrapped network^25^. As depicted in Fig. 5A, trehalose treatment on AFP-immobilized-on-Al surfaces was performed by incubating the surfaces in a trehalose-loaded solution for 1 h. When the ABP-*Cn*-AFP_G124Y_ was incubated at room temperature for up to 12 days after immobilization on Al, its denaturation was monitored via colorimetric analysis; the surface not treated with trehalose showed a rapid reduction in color (left panel in Fig. 5B and black bar in Fig. 5C). The colorimetric signal gradually decreased and the coloration declined to 34% at day 12, compared to the initial signal intensity at day 0 (Fig. 5B). It is likely that the His_6_-tagged regions of ABP-*Cn*-AFPs are susceptible to a rapid conformational change upon binding to Al, probably due to protein denaturation on the Al surface. Mass spectrometric surface analysis of amino acids revealed that histidine-rich regions of both ABP-*Cn*-AFPs disappeared after 30 days on the Al surface (data not shown). In contrast, color remained stable in the presence of trehalose over the tested time period (right panel in Fig. 5B and white bar in Fig. 5C). This result suggests that trehalose coating effectively inhibits AFP denaturation on the Al surface, and this method may be suitable for preserving the activity of surface-immobilized AFPs.

**Figure 6:**
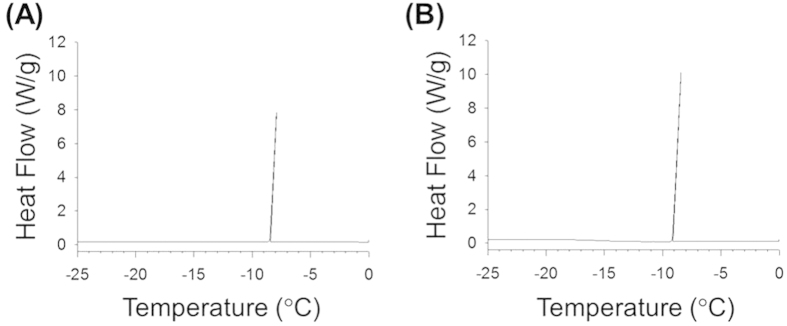
Supercooling points of ABP-*Cn*-AFP_G124Y_ on Al with and without trehalose coating. With trehalose coating, the supercooling point was −7.92 °C (**A**), and was −8.43 °C without trehalose coating (**B**) The temperature gap was 0.51 °C. Surface density of ABP-*Cn*-AFP_G124Y_ immobilized on Al was 0.04 pmol/mm^2^.

**Figure 7:**
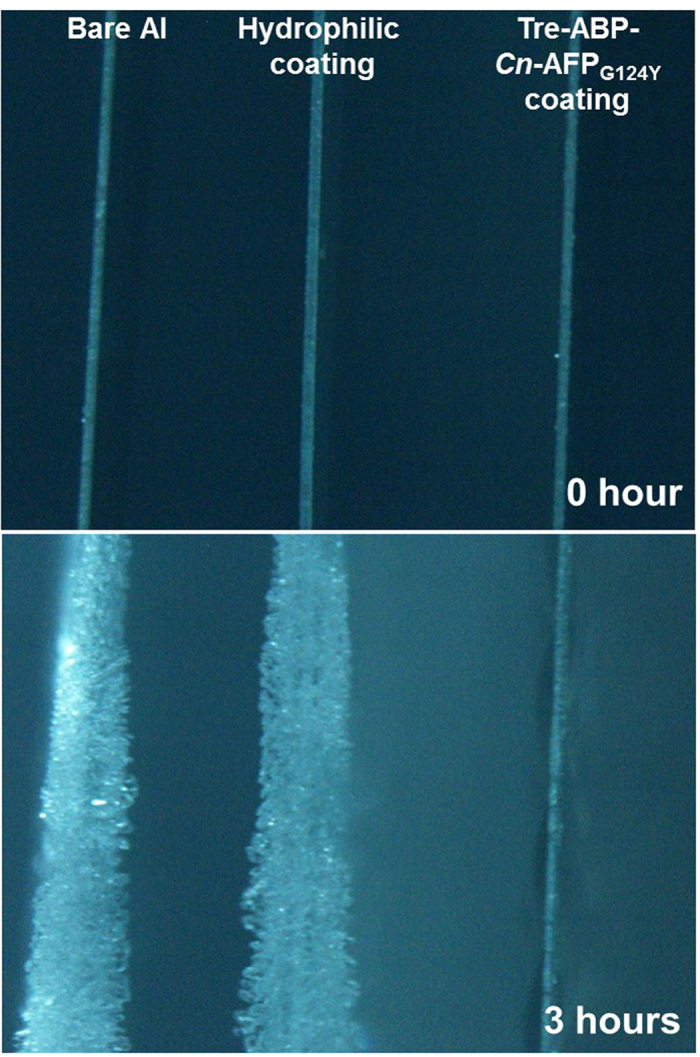
The anti-icing effect of Tre-ABP-*Cn*-AFP_G124Y_ on Al. The ABP-*Cn*-AFP_G124Y_ coating fortified by trehalose inhibited ice crystal growth and impeded frost formation, while large amounts of ice and frost accumulated on bare Al and on Al with a hydrophilic ZrO_2_ coating.

To investigate the effect of trehalose on the anti-icing activity of ABP-*Cn*-AFP, the supercooling point of ABP-*Cn*-AFP_G124Y_ was measured via differential scanning calorimetry both with and without trehalose treatment of the Al surface. Figure 6 shows a representative thermogram of the ABP-*Cn*-AFP_G124Y_ with and without trehalose on Al cooled at 0.1 °C min^−1^ from 0 °C to −25 °C. The supercooling point of ABP-*Cn*-AFP_G124Y_ in the presence of trehalose (−7.92 °C, Fig. 6A) is similar to that in the absence of trehalose (−8.43 °C, Fig. 6B). This result indicates that trehalose treatment did not affect the anti-icing activity of ABP-*Cn*-AFP_G124Y_. To further explore the possibility that directly immobilized AFP on Al prevents ice or frost formation, we examined time-dependent ice formation in a cold chamber for 3 h. Notably, *Cn*-AFP-immobilized on the Al surface (ABP-*Cn*-AFP_G124Y_ with trehalose) prevented ice formation, whereas ice formed on bare Al and hydrophilic Al surfaces coated with a thin ZrO_2_ film, which is a universal surface-coating method for Al^26^,^27^,^28^,^29^,^30^ (Fig. 7). This result demonstrates that direct immobilization of ABP-*Cn*AFP_G124Y_ followed by trehalose treatment inhibits ice formation on an Al surface.

## Discussion

The present study proposes an environmentally-friendly approach to anti-icing or anti-frosting through the coating of metals with AFP, which is further fortified by trehalose. Ice or frost formation causes serious economic and safety issues in various applications^31^ leading to burst power lines, shortened lifespans in aircraft wings, and the prevention of air circulation in refrigerators and freezers^32^. Ice problems can be solved via traditional hydrophilic polymer coatings incorporated with BaO_2_ or ZrO_2_, or via other cryoprotectant coatings such as AFPs, sugars, and polyols. AFPs have been used in cryosurgery, the storage and fermentation of foods, prevention of cellular or tissue damage via freeze/thaw cycles, and to increase the storage time of red blood cells and oocytes^6^,^10^,^33^,^34^,^35^,^36^. Despite the study of potential applications for AFPs, a practical one-step method to coat metal surfaces with AFPs for industrial applications has not been available to date, and is reported in this study for the first time.

A method of coating glass surfaces with AFPs using a chemical polymer as a protein conjugator was previously reported^19^; however, this method requires a series of complicated reaction steps and the stability of the resulting protein coating has not been verified. Therefore, we developed a one-step method to coat aluminum (which is universally used in industrial fields) with AFPs. We used mutant AFP (*Cn*-AFP_G124Y_), because it is 1.5 times more efficient in lowering freezing point^16^ than the wild-type protein, *Cn*-AFP_WT_. To immobilize the AFP on Al, we used an Al-binding peptide (ABP). The ABP did not considerably decrease *Cn*-AFP activities (Figs 1 and 2) and allowed *Cn*-AFP binding to Al (Fig. 3). Other metal-binding peptides such as those binding to silver, gold, gallium arsenide, and mild steel^22^,^37^,^38^,^39^ can also be used for the protein immobilization of respective metal surfaces. Development of fusion proteins consisting of AFPs and other metal-binding peptides could enable numerous industrial applications of AFPs.

Proteins can be easily denatured via temperature^40^. Indeed, the *Cn*-AFPs immobilized on Al were partially denatured 6 days after coating (Fig. 5B). When used in industrial applications, the denaturation of AFPs must be prevented and their stability maintained. Since trehalose is known to prevent protein denaturation and to stabilize protein structures under freezing conditions via the formation of multiple hydrogen bonds between the hydroxyl groups of trehalose and polar residues in proteins^20^,^41^, we performed trehalose coating of Al coated with *Cn*-AFP. In addition to measuring the TH activity, we measured the supercooling points of AFP proteins to further analyze their anti-icing function (Fig. 2). Mutant AFP considerably lowered the supercooling point of solution in comparison with water, clearly confirming the previously-reported TH value of *Cn-*AFP_G124Y_ in comparison with the wild-type protein^16^. Additional trehalose coating dramatically increased the stability of the immobilized protein (Fig. 5B,C). The supercooling point of ABP-*Cn*-AFP_G124Y_-Al was not considerably affected by trehalose coating (Fig. 6). Thus, trehalose coating clearly improves protein durability on metal surfaces. Frost and ice were not formed on the surface of a Tre-ABP-*Cn*-AFP_G124Y_-Al, even though large amounts of ice accumulated on the surface of a traditional hydrophilic-coated Al (Fig. 7).

In summary, our newly-developed AFP coating on Al fortified with trehalose offers remarkable benefits and advantages for the industrial application of AFPs. First, recombinant ABP-*Cn*-AFP proteins are viable for production on an industrial scale. This is an environmentally-friendly system because the production of recombinant proteins is bio-based. Secondly, Al surface coating with AFPs is accomplished rapidly via a one step-dipping method without complicated surface modification. ABP-fused AFPs are capable of maintaining the appropriate orientation of the AFP on a surface, which allows for application of their full anti-icing properties. Most importantly, AFPs clearly impeded ice formation on the Al surface when compared to bare Al and traditional hydrophilic Al coatings. This effect was due to the TH activity of *Cn*-AFP and its ability to lower the freezing point. The results of this study will provide numerous opportunities for applications in the cryostat, refrigerator, and freezer industries to protect against frost and ice formation.

## Methods

### Biochemical reagents

The following reagents were used: D-(+)-trehalose dehydrate from *Saccharomyces cerevisiae* (>99%, Sigma-Aldrich), 3, 3′, 5, 5′-tetramethylbenzidine (1-Step Turbo TMB, Thermo Scientific, U.S.A.), and HisProbe-horseradish peroxidase conjugate (HisProbe-HRP, Thermo Scientific, U.S.A.).

### Construction and purification of *Cn*-AFP and Al-binding peptide (ABP)-fused *Cn*-AFP

To prepare an expression construct to produce the ABP-*Cn*-AFP fusion protein, the 5′-forward primer encoding the aluminum-binding peptide (VPSSGPQDTRTT; shown in bold in Table 1) was used. The 5′- and 3′-primers included the *Xho*I and *Sal*I restriction sites, respectively (underlined in Table 1). The open reading frame (ORF) encoding the active form of *Cn*-AFP (without a signal peptide) was amplified by PCR using the genomic DNA of *C. neogracile*, and was used to produce *Cn*-AFP_G124Y_ via site-directed mutagenesis, as described in our previous report^16^. *Cn*-AFP genes were amplified by PCR, digested with *Xho*I and *Sal*I, and ligated into the pColdI expression vector (Takara, Japan). The expression vectors were transformed into *E. coli* BL21 (DE3), and the pColdI expression method (Takara, Japan) was used to induce protein production. Cells were disrupted by sonication and soluble recombinant proteins were purified by His-tag affinity chromatography (Qiagen, U.S.A)^16^. Purified proteins were concentrated by Centricon microconcentrators (Millipore, U.S.A.) and the protein concentration was measured using the Bradford reagent (BioRad, U.S.A.).

### AFP immobilization on Al

An Al substrate 1 mm in thickness was cut into 10 mm × 10 mm sections and cleaned via subsequent immersion in the following solutions: i) 10% Na_2_CO_3_ (pH 11) for 1–2 min at 40–45 °C, ii) deionized water (washing three times), iii) 5–10% NaOH (pH 11) for 1–2 min at RT, and iv) deionized water (washing three times). The substrates were then dried in air prior to use. For the formation of an AFP-coated surface, Al substrates were immersed in a 10 μM solution containing either *Cn*-AFP_G124Y_ or ABP-*Cn*-AFP_G124Y_ fusion proteins (dissolved in 10 mM phosphate-buffered saline (PBS), pH 7.4) for 2 h at RT Al surface, followed by thorough rinsing with distilled water (three times). To minimize protein denaturation, each AFP-coated Al substrate was immersed in 0.1% (w/v) trehalose solution in 10 mM PBS (pH 7.4) for 1 h at RT in a conventional 12-well plate. Trehalose-treated substrates were then air-dried prior to use.

### Characterization of surface-immobilized AFPs on an Al surface

For direct detection of the His_6_-tag at its N-terminal region of the AFP or ABP-AFP fusion proteins on Al, protein-coated Al surfaces were immersed in a nickel-activated HisProbe-HRP solution (at a final concentration of 1 μg mL^−1^ dissolved in 100 mM PBS containing 0.01% Tween-20, pH 7.2) for 2 h at RT, followed by thorough rinsing with a washing buffer (100 mM PBS containing 0.01% Tween-20, pH 7.2) (three times), according to the manufacturer’s instructions. The surfaces were then immersed in a 1 mL stock solution of 1-Step Turbo TMB (Thermo, U.S.A.) as a soluble colorimetric substrate for HRP. After 30–60 min of incubation at RT, an aliquot (100 μL) from the reactant blue solution was transferred to a new 96-well plate. The absorbance spectrum of the solution was measured using a micro-plate reader equipped with a UV-spectrophotometer (Varioskan Flash Spectral Scanning Multimode Reader, Thermo, U.S.A.). The absorption maxima were observed at 370 nm, as defined elsewhere^42^.

To further verify the AFP immobilization on Al, surface infrared (IR) analysis was performed in attenuated total reflectance mode using a Fourier transform (FT)-IR spectrophotometer (IFS66V/S & Hyperion 3000, Bruker Optiks, Germany) equipped with the Smart Apertured Grazing Angle (SAGA) accessory. A total of 64 scans on average were performed to yield the IR spectrum at a resolution of 4 cm^−1^. All spectra of ABP-*Cn*-AFP_WT_-Al and control surfaces (bare Al and *Cn*-AFP_WT_-Al) were displayed in the absorption mode, and ranged from 4,000 to 400 cm^−1^.

### Characterization of the anti-icing activity of AFPs immobilized on Al

The supercooling points of an Al substrate coated with ABP-*Cn*-AFP_G124Y_ and an Al substrate coated with Tre-ABP-*Cn*-AFP_G124Y_ were measured using a differential scanning calorimeter (DSC Q100; TA instruments, U.S.A.). The temperature of the sample stage was lowered by 0.1 °C min^−1^ from 0 °C to −25 °C. The antifreeze activities of the fusion proteins (5 mg/mL) were assayed on the basis of thermal hysteresis and ice crystal morphology using a nanoliter osmometer (Otago Osmometer, New Zealand). Briefly, the recombinant proteins were placed at the sample stage, and the temperature of the sample stage was lowered to a rate of 0.01 °C min^−1^. During cooling, the TH values were measured and the ice crystals were observed under a light microscope equipped with a CCD camera (BX53, Olympus, Japan). The anti-icing activity of *Cn*-AFP-Al was observed in an isothermal-isohumidity chamber. The temperature and relative humidity of the chamber were maintained at −3.5 °C and 84%, respectively, while air (flow velocity, 0.5 m s^−1^) and a refrigerant (brine; flow velocity 2 L min^−1^; −12 °C) flowed for 3 h. Tre-ABP-*Cn*-AFP_G124Y_-Al, bare Al, and Al with hydrophilic ZrO_2_ coating were used in these experiments.

## Additional Information

**How to cite this article**: Gwak, Y. *et al.* Creating Anti-icing Surfaces via the Direct Immobilization of Antifreeze Proteins on Aluminum. *Sci. Rep.* **5**, 12019; doi: 10.1038/srep12019 (2015).
